# 4-(*p*-Tolyl­amino)­benzaldehyde

**DOI:** 10.1107/S1600536810040389

**Published:** 2010-10-20

**Authors:** Xiao Tian, Yong-Sheng Xie, Hua Zuo

**Affiliations:** a College of Pharmaceutical Sciences, Southwest University, Chongqing 400715, People’s Republic of China; b School of Chemical and Environmental Engineering, Chongqing Three Gorges University, Chongqing 404100, People’s Republic of China

## Abstract

In the title compound, C_14_H_13_NO, the dihedral angle between the aromatic rings is 66.08 (9)°. Chains are formed along the *b* axis through inter­molecular N—H⋯O hydrogen bonds. The crystal structure is further stabilized by C—H⋯π inter­actions.

## Related literature

For applications and bioactivity of diaryl­amines, see: Abou-Seri (2010 ▶); Kostrab *et al.* (2008 ▶).
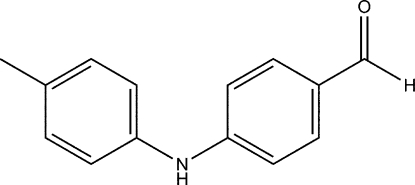


## Experimental

### 

#### Crystal data

C_14_H_13_NO
*M* *_r_* = 211.25
Orthorhombic, 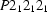

*a* = 5.8356 (12) Å
*b* = 8.2581 (18) Å
*c* = 24.137 (5) Å
*V* = 1163.2 (4) Å^3^
*Z* = 4
Mo *K*α radiation
μ = 0.08 mm^−1^
*T* = 298 K
0.20 × 0.20 × 0.10 mm

#### Data collection

Bruker SMART area-detector diffractometer
6780 measured reflections
1555 independent reflections
1414 reflections with *I* > 2σ(*I*)
*R*_int_ = 0.021

#### Refinement

*R*[*F* ^2^ > 2σ(*F* ^2^)] = 0.038
*wR*(*F* ^2^) = 0.110
*S* = 1.07
1555 reflections
153 parameters
H atoms treated by a mixture of independent and constrained refinement
Δρ_max_ = 0.12 e Å^−3^
Δρ_min_ = −0.16 e Å^−3^

### 

Data collection: *SMART* (Bruker, 2004 ▶); cell refinement: *SAINT* (Bruker, 2004 ▶); data reduction: *SAINT*; program(s) used to solve structure: *SHELXTL* (Sheldrick, 2008 ▶); program(s) used to refine structure: *SHELXTL*; molecular graphics: *SHELXTL*; software used to prepare material for publication: *SHELXTL*.

## Supplementary Material

Crystal structure: contains datablocks I, global. DOI: 10.1107/S1600536810040389/zl2311sup1.cif

Structure factors: contains datablocks I. DOI: 10.1107/S1600536810040389/zl2311Isup2.hkl

Additional supplementary materials:  crystallographic information; 3D view; checkCIF report

## Figures and Tables

**Table 1:** Hydrogen-bond geometry (Å, °) *Cg*1 is the centroid of the C8–C13 ring.

*D*—H⋯*A*	*D*—H	H⋯*A*	*D*⋯*A*	*D*—H⋯*A*
N1—H1⋯O2^i^	0.84 (3)	2.11 (3)	2.934 (2)	167 (2)
C1—H1*A*⋯*Cg*1^ii^	0.96	2.91	3.613 (2)	131
C12—H12⋯*Cg*1^iii^	0.93	2.77	3.527 (2)	140

Symmetry codes: (i) 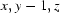; (ii) 
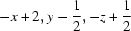
; (iii) 
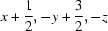
.

**Fig. 1.**
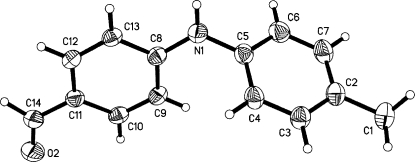
The molecular structure of the title compound, with displacement ellipsoids drawn at the 30% probability level.

**Fig. 2.**
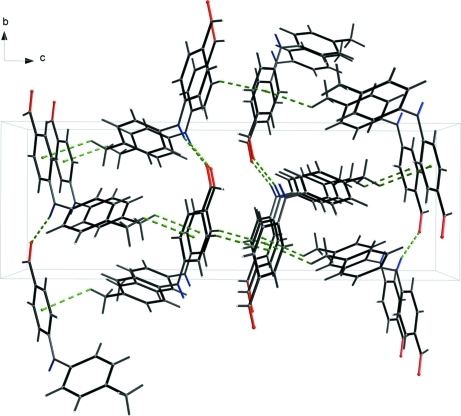
The crystal packing of the title compound. Intermolecular hydrogen bonds and C—H···π contacts are shown as dashed lines.

## supplementary crystallographic information

### Comment

Due to their wide range of applications and special pharmacological activities diarylamines represent an important class of chemical compounds (Abou-Seri, 2010; Kostrab *et al.,* 2008). We report here the synthesis and the crystal structure of one such diarylamine, the title compound 4-(p-tolylamino)benzaldehyde.

The title compound, C_14_H_13_NO, consists of benzaldehyde and tolyl groups connected through a central amino nitrogen atom (Fig. 1). The dihedral angle between the aromatic rings is 66.08 (9)°. In the crystal, one-dimensional chains are formed along the *b*-axis through intermolecular N—H···O hydrogen bonding interactions (Fig. 2). The chains are in turn linked through weak intermolecular C—H···π contacts involving the C8–C13 phenyl ring (centroid Cg1) into a three-dimensional network structure (Table 1).

### Experimental

To a magnetically stirred solution of *p*-toluidine (1.0 mmol) and Cs_2_CO_3_ (3.2 mmol) in dry DMF cooled by an ice bath were added chloroacetyl chloride (1.2 mmol) and 4-hydroxybenzaldehyde (1.0 mmol). The reaction mixture was then stirred for 30 min at room temperature and placed into a microwave oven (600 W, 424 K) and irradiated for 35 min. The solvent was removed under vacuum and water (20 ml) was added to the residue. The mixture was then extracted with ethyl acetate (4 × 30 ml). The combined organic layers were dried over anhydrous MgSO_4_ and evaporated under vacuum to give the crude product. The product was purified by column chromatography on silica gel using ethyl acetate/petroleum ether (yield 89%). Mp 358-362 K; ^1^H NMR (300 MHz, CDCl_3_): δ 2.35 (s, 3H; CH_3_), 6.20 (s, 1H; NH), 6.95 (d, *J*  = 8.4 Hz, 2H; ArH), 7.10 (d, *J*  = 8.1 Hz, 2H; ArH), 7.18 (d, *J*  = 8.1 Hz, 2H; ArH), 7.72 (d, *J*  = 8.4 Hz, 2H; ArH), 9.77 (s, 1H; CHO). ^13^C NMR (75 MHz, CDCl_3_): δ 20.8 (CH_3_), 113.9 (CH), 122.1 (CH), 128.1 (C), 130.1 (CH), 132.1 (CH), 134.0 (C), 137.2 (C), 150.5 (C), 190.3 (CHO). Crystals suitable for X-ray diffraction were obtained by slow evaporation of a solution of the solid dissolved in ethyl acetate/petroleum ether at room temperature for 5 days.

### Refinement

In the absence of significant anomalous dispersion effects, Friedel pairs were merged.

Aromatic and methyl H atoms were positioned geometrically and refined using a riding model, with C—H = 0.93 Å for aryl and 0.96 Å for methyl H atoms, and with *U*_iso_(H) =1.2*U*_eq_(C) for aryl Hatoms, and 1.5*U*_eq_(C) for methyl H atoms. The aldehyde (C14) and N-bound H-atoms were located in a difference Fourier map and their positions and *U*_iso_ values were freely refined.

### Crystal data

**Table:**

C_14_H_13_NO	*D*_x_ = 1.206 Mg m^−^^3^
*M**_r_* = 211.25	Mo *K*α radiation, λ = 0.71073 Å
Orthorhombic, *P*2_1_2_1_2_1_	Cell parameters from 3865 reflections
*a* = 5.8356 (12) Å	θ = 2.6–27.0°
*b* = 8.2581 (18) Å	µ = 0.08 mm^−^^1^
*c* = 24.137 (5) Å	*T* = 298 K
*V* = 1163.2 (4) Å^3^	Block, brown
*Z* = 4	0.20 × 0.20 × 0.10 mm
*F*(000) = 448	

### Data collection

**Table:**

Bruker SMART area-detector diffractometer	1414 reflections with *I* > 2σ(*I*)
Radiation source: fine-focus sealed tube	*R*_int_ = 0.021
graphite	θ_max_ = 27.4°, θ_min_ = 2.6°
phi and ω scans	*h* = −7→7
6780 measured reflections	*k* = −10→7
1555 independent reflections	*l* = −31→24

### Refinement

**Table:**

Refinement on *F*^2^	Primary atom site location: structure-invariant direct methods
Least-squares matrix: full	Secondary atom site location: difference Fourier map
*R*[*F*^2^ > 2σ(*F*^2^)] = 0.038	Hydrogen site location: inferred from neighbouring sites
*wR*(*F*^2^) = 0.110	H atoms treated by a mixture of independent and constrained refinement
*S* = 1.07	*w* = 1/[σ^2^(*F*_o_^2^) + (0.0662*P*)^2^ + 0.0847*P*] where *P* = (*F*_o_^2^ + 2*F*_c_^2^)/3
1555 reflections	(Δ/σ)_max_ < 0.001
153 parameters	Δρ_max_ = 0.12 e Å^−^^3^
0 restraints	Δρ_min_ = −0.16 e Å^−^^3^

### Special details

**Table:**

Geometry. All e.s.d.'s (except the e.s.d. in the dihedral angle between two l.s. planes) are estimated using the full covariance matrix. The cell e.s.d.'s are taken into account individually in the estimation of e.s.d.'s in distances, angles and torsion angles; correlations between e.s.d.'s in cell parameters are only used when they are defined by crystal symmetry. An approximate (isotropic) treatment of cell e.s.d.'s is used for estimating e.s.d.'s involving l.s. planes.
Refinement. Refinement of *F*^2^ against ALL reflections. The weighted *R*-factor *wR* and goodness of fit *S* are based on *F*^2^, conventional *R*-factors *R* are based on *F*, with *F* set to zero for negative *F*^2^. The threshold expression of *F*^2^ > σ(*F*^2^) is used only for calculating *R*-factors(gt) *etc*. and is not relevant to the choice of reflections for refinement. *R*-factors based on *F*^2^ are statistically about twice as large as those based on *F*, and *R*- factors based on ALL data will be even larger.

### Fractional atomic coordinates and isotropic or equivalent isotropic displacement parameters (Å^2^)

**Table:**

	*x*	*y*	*z*	*U*_iso_*/*U*_eq_	
C13	1.3763 (3)	0.6409 (2)	0.06501 (7)	0.0509 (4)	
H13	1.4615	0.5491	0.0564	0.061*	
C11	1.3240 (3)	0.9306 (2)	0.05882 (6)	0.0506 (4)	
C8	1.1747 (3)	0.6263 (2)	0.09648 (6)	0.0490 (4)	
C12	1.4480 (3)	0.7908 (2)	0.04698 (6)	0.0502 (4)	
H12	1.5823	0.7988	0.0264	0.060*	
C5	0.9454 (3)	0.4459 (2)	0.15636 (7)	0.0537 (4)	
C10	1.1209 (3)	0.9158 (2)	0.08968 (7)	0.0536 (4)	
H10	1.0348	1.0077	0.0976	0.064*	
N1	1.1016 (3)	0.4765 (2)	0.11257 (7)	0.0638 (5)	
C9	1.0479 (3)	0.7677 (2)	0.10832 (7)	0.0530 (4)	
H9	0.9137	0.7603	0.1289	0.064*	
C3	0.8304 (3)	0.4783 (2)	0.25130 (7)	0.0588 (5)	
H3	0.8561	0.5239	0.2860	0.071*	
C2	0.6412 (3)	0.3791 (2)	0.24364 (8)	0.0568 (4)	
C14	1.4066 (4)	1.0868 (2)	0.03930 (8)	0.0650 (5)	
C6	0.7577 (4)	0.3455 (3)	0.14822 (9)	0.0667 (5)	
H6	0.7321	0.2995	0.1136	0.080*	
C7	0.6087 (4)	0.3136 (3)	0.19139 (9)	0.0682 (5)	
H7	0.4834	0.2464	0.1852	0.082*	
C4	0.9812 (3)	0.5110 (2)	0.20869 (8)	0.0587 (5)	
H4	1.1076	0.5770	0.2151	0.070*	
C1	0.4755 (4)	0.3446 (3)	0.29038 (9)	0.0733 (6)	
H1A	0.5256	0.3995	0.3233	0.110*	
H1B	0.4702	0.2301	0.2972	0.110*	
H1C	0.3255	0.3823	0.2803	0.110*	
O2	1.3205 (4)	1.21695 (17)	0.04864 (7)	0.0910 (6)	
H1	1.162 (5)	0.393 (3)	0.0987 (10)	0.076 (7)*	
H14	1.551 (5)	1.074 (3)	0.0161 (11)	0.087 (7)*	

### Atomic displacement parameters (Å^2^)

**Table:**

	*U*^11^	*U*^22^	*U*^33^	*U*^12^	*U*^13^	*U*^23^
C13	0.0529 (9)	0.0508 (9)	0.0490 (8)	0.0106 (8)	0.0053 (7)	−0.0020 (7)
C11	0.0570 (9)	0.0502 (9)	0.0446 (7)	0.0004 (8)	0.0003 (7)	−0.0041 (7)
C8	0.0523 (8)	0.0529 (8)	0.0419 (7)	0.0040 (8)	0.0004 (7)	0.0004 (7)
C12	0.0486 (8)	0.0565 (9)	0.0454 (8)	0.0030 (8)	0.0047 (7)	−0.0030 (7)
C5	0.0563 (9)	0.0489 (8)	0.0558 (9)	0.0009 (9)	0.0032 (8)	0.0065 (7)
C10	0.0582 (10)	0.0513 (9)	0.0514 (8)	0.0125 (8)	0.0035 (8)	−0.0045 (7)
N1	0.0748 (11)	0.0499 (8)	0.0666 (10)	0.0029 (9)	0.0189 (9)	0.0005 (7)
C9	0.0491 (8)	0.0585 (9)	0.0513 (8)	0.0074 (8)	0.0073 (8)	−0.0002 (7)
C3	0.0582 (9)	0.0669 (11)	0.0514 (9)	−0.0040 (9)	−0.0042 (8)	0.0070 (8)
C2	0.0509 (9)	0.0592 (10)	0.0603 (10)	0.0016 (9)	−0.0016 (8)	0.0133 (8)
C14	0.0802 (14)	0.0515 (10)	0.0634 (10)	−0.0061 (11)	0.0099 (11)	−0.0073 (8)
C6	0.0773 (13)	0.0638 (11)	0.0591 (10)	−0.0120 (11)	−0.0060 (10)	−0.0020 (9)
C7	0.0612 (11)	0.0691 (12)	0.0744 (11)	−0.0182 (11)	−0.0038 (10)	0.0073 (10)
C4	0.0516 (9)	0.0642 (10)	0.0602 (10)	−0.0113 (9)	−0.0036 (8)	0.0056 (8)
C1	0.0611 (11)	0.0859 (14)	0.0727 (12)	−0.0038 (12)	0.0070 (10)	0.0197 (11)
O2	0.1203 (14)	0.0497 (8)	0.1029 (12)	0.0005 (10)	0.0241 (11)	−0.0093 (8)

### Geometric parameters (Å, °)

**Table:**

C13—C12	1.377 (3)	C9—H9	0.9300
C13—C8	1.406 (3)	C3—C4	1.380 (3)
C13—H13	0.9300	C3—C2	1.387 (3)
C11—C12	1.392 (2)	C3—H3	0.9300
C11—C10	1.405 (3)	C2—C7	1.385 (3)
C11—C14	1.455 (3)	C2—C1	1.513 (3)
C8—N1	1.365 (2)	C14—O2	1.208 (3)
C8—C9	1.411 (2)	C14—H14	1.02 (3)
C12—H12	0.9300	C6—C7	1.383 (3)
C5—C6	1.388 (3)	C6—H6	0.9300
C5—C4	1.388 (3)	C7—H7	0.9300
C5—N1	1.419 (2)	C4—H4	0.9300
C10—C9	1.371 (2)	C1—H1A	0.9600
C10—H10	0.9300	C1—H1B	0.9600
N1—H1	0.84 (2)	C1—H1C	0.9600
			
C12—C13—C8	120.13 (16)	C4—C3—C2	121.58 (17)
C12—C13—H13	119.9	C4—C3—H3	119.2
C8—C13—H13	119.9	C2—C3—H3	119.2
C12—C11—C10	118.36 (16)	C7—C2—C3	117.45 (17)
C12—C11—C14	119.75 (17)	C7—C2—C1	121.18 (18)
C10—C11—C14	121.89 (17)	C3—C2—C1	121.36 (18)
N1—C8—C13	119.53 (16)	O2—C14—C11	126.2 (2)
N1—C8—C9	121.91 (16)	O2—C14—H14	122.7 (14)
C13—C8—C9	118.50 (16)	C11—C14—H14	111.1 (14)
C13—C12—C11	121.52 (16)	C7—C6—C5	120.24 (19)
C13—C12—H12	119.2	C7—C6—H6	119.9
C11—C12—H12	119.2	C5—C6—H6	119.9
C6—C5—C4	118.60 (16)	C6—C7—C2	121.70 (19)
C6—C5—N1	120.55 (17)	C6—C7—H7	119.1
C4—C5—N1	120.81 (17)	C2—C7—H7	119.1
C9—C10—C11	120.89 (16)	C3—C4—C5	120.41 (17)
C9—C10—H10	119.6	C3—C4—H4	119.8
C11—C10—H10	119.6	C5—C4—H4	119.8
C8—N1—C5	125.06 (16)	C2—C1—H1A	109.5
C8—N1—H1	119.7 (17)	C2—C1—H1B	109.5
C5—N1—H1	114.8 (17)	H1A—C1—H1B	109.5
C10—C9—C8	120.59 (16)	C2—C1—H1C	109.5
C10—C9—H9	119.7	H1A—C1—H1C	109.5
C8—C9—H9	119.7	H1B—C1—H1C	109.5

### Hydrogen-bond geometry (Å, °)

**Table:**

Cg1 is the centroid of the C8–C13 ring.

**Table:**

*D*—H···*A*	*D*—H	H···*A*	*D*···*A*	*D*—H···*A*
N1—H1···O2^i^	0.84 (3)	2.11 (3)	2.934 (2)	167 (2)
C1—H1A···Cg1^ii^	0.96	2.91	3.613 (2)	131
C12—H12···Cg1^iii^	0.93	2.77	3.527 (2)	140

Symmetry codes: (i) *x*, *y*−1, *z*; (ii) −*x*+2, *y*−1/2, −*z*+1/2; (iii) *x*+1/2, −*y*+3/2, −*z*.
